# A metabolic-inflammatory-nutritional score (MINS) is associated with lymph node metastasis and prognostic stratification for endometrial cancer patients

**DOI:** 10.7150/ijms.96179

**Published:** 2024-09-09

**Authors:** Xite Lin, Tianai Chen, Liang Wang, Yuan Ren, Wenyu Lin, Xiaodan Mao, Pengming Sun

**Affiliations:** 1 Laboratory of Gynecologic Oncology, Fujian Maternity and Child Health Hospital, College of Clinical Medicine for Obstetrics & Gynecology and Pediatrics, Fujian Medical University, Fuzhou 350001, China.; 2 Fujian Key Laboratory of Women and Children's Critical Diseases Research, Fujian Maternity and Child Health Hospital, Fuzhou 350001, China.; 3 Fujian Clinical Research Center for Gynecological Oncology, Fujian Maternity and Child Health Hospital, Fuzhou 350001, China.; 4 Department of Obstetrics and Gynecology, Fujian Maternity and Child Health Hospital, Affiliated Hospital of Fujian Medical University, Fuzhou 350001, China.

**Keywords:** Endometrial cancer, Lymph node metastasis, Metabolism, Inflammation, Nutrition

## Abstract

**Objective:** This study aims to propose a personalized cancer prediction model based on the metabolic-inflammatory-nutritional score (MINS) for predicting lymph node metastasis (LNM) in endometrial cancer (EC) and validated prediction of survival probability in patients with a family history of Lynch syndrome-associated cancers (LSAC).

**Methods:** A total of 676 patients diagnosed with EC were enrolled in this study. We calculated the optimal cutoff value using restricted cubic splines (RCS) analysis or the mean value. Our feature selection process for constructing the MINS involved using the LASSO regression model. MINS were evaluated for LNM using logistic regression analysis. To assess the prognostic value of the MINS, we generated survival curves using the Kaplan-Meier method with a log-rank test. Furthermore, we constructed a nomogram to validate the prognostic significance of the MINS. The predictive accuracy of nomogram was evaluated using the concordance index (C-index) and calibration plot.

**Results:** LNM risk was associated with family history of LSAC and MINS group (all adjusted p<0.05). Patients in the high-risk MINS group or patients with a family history of LSAC exhibited poorer overall survival (p=0.038, p=0.001, respectively). Additionally, a nomogram was demonstrated effective predictive performance with a C-index of 0.778 (95% CI: 0.725-0.832).

**Conclusion:** Preoperative MINS has been determined to be associated with the risk of LNM in EC patients. Utilizing MINS as a basis, the development of a prognostic nomogram holds promise as an effective tool for risk stratification in clinical settings among EC patients with a family history of LSAC.

## 1. Introduction

Endometrial cancer (EC) is a prevalent type of gynecologic cancers that has been on the rise globally in recent decades 1. The continued increase in incidence and disease-related mortality rates of EC underscores the importance of strategies for early detection and prevention 2, 3. While many cases of EC are detected at an early stage, leading to a favorable prognosis, a significant number of women are diagnosed with advanced or metastatic disease, which unfortunately carries a bleak outlook 4. Lymph node metastasis (LNM) is widely recognized as the most significant prognostic factor in EC. Patients with LNM face a higher risk of recurrence and overall poorer survival compared to those without LNM 5. There has been extensive research in recent years investigating lymph node dissection's possible role in EC, but controversy remains regarding the indications, the anatomic extent, and the therapeutic benefits. Routine lymphadenectomy ensures accurate staging and determines appropriate postoperative treatment, but does not improve disease-free or overall survival. Instead, it is associated with an increased incidence of surgery-related complications 6. However, complications that may arise from lymphadenectomy include lymphocyst formation, deep-vein thrombosis, postsurgical lymphedema, and endothelial and neurovascular injury 7. To better inform surgical planning and discussions about prognosis and the appropriate surgical approach with patients, it would be valuable to have an understanding of which patients are at a higher risk of preoperative lymph node involvement.

As mentioned in the literature review, chronic inflammation, metabolic and nutritional dysregulation are closely associated with tumorigenesis and progression and are critical to patient survival and prognosis 8-10. Moreover, there exists a profound interconnectedness between metabolism, nutrition, inflammation and cancer, with each element having a profound impact on the others 11. A surplus consumption of nutrients can lead to metabolic disorders, which are triggered by a pervasive chronic inflammation that is also referred to as metabolic inflammation 12.

Currently, many biomarkers based on peripheral blood cell analysis and biochemical tests show promise in guiding the clinical management of cancer patients across different malignancies. Previous studies have demonstrated that a low controlling nutritional status (CONUT) score 13, prognostic nutritional index (PNI) score and monocyte-to-lymphocyte ratio (MLR) are associated with favorable prognosis, and an elevated preoperative neutrophil-to-lymphocyte ratio (NLR), platelet-to-lymphocyte ratio (PLR) level worsens OS in patients with EC 14, 15. Recent studies have shown that triglycerides-to-high density lipoprotein cholesterol ratio (TG/HDL-C) and triglyceride-glucose index (TyG) independently predict EC risk 16, 17. Prior work from our group demonstrated that preoperative systemic immune-inflammatory index (SII) could assist in the prediction of LNM among EC patients^18^. These assessment tools for metabolism, nutrition, and inflammation offer numerous clinical advantages, including ease of implementation, accessibility during the perioperative period, and low cost. Preoperative biomarkers in peripheral blood alone can't reflect the landscape of a patient's metabolic profile, nutrition status, or inflammation levels. In terms of overcoming this limitation, we believe that integrating these markers may provide more accurate prognostic information and be more meaningful than relying on individual indicators alone. So far, few studies investigated the prediction model based on the combination of metabolism factors, systemic inflammation and nutritional status in EC. Therefore, a new statistical model is necessary that synthesizes the biological indicators.

Lynch syndrome (LS) is a hereditary cancer syndrome that is characterized by an increased risk of developing tumors in various parts of the body. EC often presents as a sentinel cancer in individuals with Lynch syndrome, and its detection can potentially lead to the prevention of secondary cancers 19. However, the relationship between a family history of Lynch syndrome-associated cancers (LSAC) and the clinical features and outcomes of EC is not well-established.

In view of this, our team proposed a personalized cancer prediction model based on the metabolic-inflammatory-nutritional system for predicting LNM in EC to identify patients with different risk of LNM and validated prediction of survival probability in a subgroup of patients with a family history of LSAC.

## 2. Materials and Methods

### 2.1. Patients and study design

The retrospective assessment involved the analysis of data from 676 EC patients who had undergone comprehensive surgical staging with pelvic lymphadenectomy at the Fujian Maternity and Child Health Hospital, Affiliated Hospital of Fujian Medical University. The study period extended from January 2013 to January 2023. The inclusion criteria included the following: (1) the patients had undergone surgical staging (hysterectomy and bilateral salpingo-oophorectomy with lymphadenectomy); (2) postoperative histologically proven EC; (3) no other treatments, such as radiotherapy, chemotherapy, or hormonal therapy, had been administered prior to surgery; (4) complete baseline laboratory test information within two weeks prior to surgery. The exclusion criteria were: (1) presence of other malignancies; (2) existence of severe diseases or infections; (3) young patients who opted for fertility preservation; (4) incomplete clinical data or lack of follow-up information. According to FIGO (International Federation of Gynecology and Obstetrics) and ESMO (European Society of Medical Oncology) guidelines, all patients were evaluated for surgical staging by EC, and surgery was performed by an experienced surgical team. All pathologic evaluations were performed by expert gynecologic pathologists, and final pathologic diagnoses were confirmed. As this was a retrospective, non-interventional and observational study in which the patient data used were kept strictly confidential, the requirement for informed consent was waived. The study received approval from the Ethics Commission of the Fujian Maternity and Child Health Hospital (approval number 2023KY140).

### 2.2. Data collection and definition of variables

The demographic and clinical characteristics were collected. The following nine types of cancers/tumors are considered LSAC: colon, gastric, endometrial, ovarian, sebaceous gland, urothelial carcinoma of the ureter, glioblastoma, prostate or breast. Family history of LSAC was defined as the history of cancer in each proband's first-degree relative (FDR; parent, sibling, child of proband). Preoperative hematological parameters (normal value), including albumin (40g/L), fasting blood glucose (6.1mmol/L); neutrophil count (6.3×10^9^/L), lymphocyte count (1.1×10^9^/L), monocyte count (0.6×10^9^/L), and platelet count (350×10^9^/L); triglyceride (1.7mmol/L), total cholesterol (5.2mmol/L), high-density lipoprotein cholesterol (1.29mmol/L), and low-density lipoprotein cholesterol (3.10 mmol/L), were grouped according to the standards developed by Fujian Maternity and Child Health Hospital's clinical laboratory standards. According to Asian-specific criteria, overweight/obesity was defined by a body mass index (BMI)≥ 23 kg/m^2^ and non-overweight/obesity was defined as BMI<23. In this study, metabolic biomarkers include TyG, TG/HDL-C, total cholesterol-to- high density lipoprotein cholesterol ratio (TC/HDL-C), low-density lipoprotein cholesterol-to-high-density lipoprotein cholesterol ratio (LDL/HDL-C). Systemic inflammation biomarkers include NLR, SII, PLR, lymphocyte-to-monocyte ratio (LMR) and glucose-to-lymphocyte ratio (GLR). Nutrition biomarkers include body mass index (BMI), PNI, nutritional risk index (NRI), and CONUT. The calculation formulas and details for each biomarker were given in the Supplementary Materials (**Table S2**). Post-operation pathological indicators were collected. These indicators included histological type, FIGO stage, histological grade, myometrium invasion (MI), cervical stromal involvement (CSI), LNM, and lymphovascular space invasion (LVSI). Lastly, the overall survival (OS) was defined as the time interval between the commencement of curative surgery and death from any cause or the last follow-up.

### 2.3. MINS construction

Restricted cubic splines (RCS) allow for potential nonlinear relationships between biomarker level and LNM. For the biomarker that meeting the nonlinear relationship criterion (p-Nonlinear < 0.05), the most appropriate cutoff value is the optimal decision threshold based on the RCS analysis. In cases where there is no evidence to support a nonlinear relationship, the cutoff values are established based on the mean values of the patients. Each covariate's variance inflation factor (VIF) was calculated to assess for collinearity. Feature selection for the construction of the MINS is carried out using the least absolute shrinkage and selection operator (LASSO) regression model. Finally, MINS is calculated based on the variables that have non-zero coefficients.

### 2.4. Statistical analysis

Statistical analysis was conducted using the R statistical language (version 4.2.2, R Foundation for Statistical Computing, Vienna, Austria) and SPSS software (IBM Corp. Released 2019. IBM SPSS Statistics for Mac OS, Version 26.0. Armonk, NY, USA: IBM Corp). Continuous variables were expressed as means ± standard deviations (SD) or medians and interquartile range (IQR) while categorical variables were presented as percentages. The "rms" R package was used for RCS analysis. RCS curves (four knots) were generated using the "ggplot2" R package, and images were processed further using the "ggprism" R package. As a means to avoid the impact of collinearity between individual biomarkers, we used LASSO regression with the "glmnet" R package, and we eliminated biomarkers with large collinearities by Pearson's test. Logistic regression analyses were used to assess associations between the MINS and LNM. The Kaplan-Meier (K-M) curves were plotted to compare differences in OS, and the log-rank test was used for statistical analysis. Cox Proportional-Hazards regression analysis was performed to identify the independent factors significantly associated with OS, with adjustments made for potential confounders. Variables with a p-value less than 0.05 in the univariate analyses were subjected to further multivariate analyses. A significance level of p < 0.05 was considered statistically significant. Based on the independent OS predictions derived from the Cox Proportional-Hazards regression analysis, a nomogram was established and graphically represented using the "rms" package in R software. Subsequently, the concordance index (C-index) and calibration curves were used to evaluate this prognostic model's accuracy and predictability.

## 3. Results

### 3.1. Basic characteristics of patients

The baseline demographic and clinical characteristics of the EC patients (n = 676) are presented in **Table S1**. The median age at diagnosis of EC was 54 years old (IQR: 50~59). Over 57.8% of the cases were post-menopause. The majority of 1060 (87.0%) patients were pathologically diagnosed with endometrioid EC, and 98 (14.5%) patients had a family history of LSAC. Furthermore, the sum of 579 (85.7%) patients were diagnosed at FIGO stage I or II, while 97 (14.3%) were diagnosed at FIGO stage III or IV. The tumor grade G1-G2, and G3 were 488(86.8%) and 74 (13.2%), respectively. Most cases showed pathological characteristics including negative CSI (81.1%), <50% MI (71.6%), negative LVSI (86.1%), negative LNM (91.4%). Serum metabolic, inflammatory, and nutritional indicators were collected and summarized in **Table S1**.

### 3.2. MINS Construction

**Figure 1:**
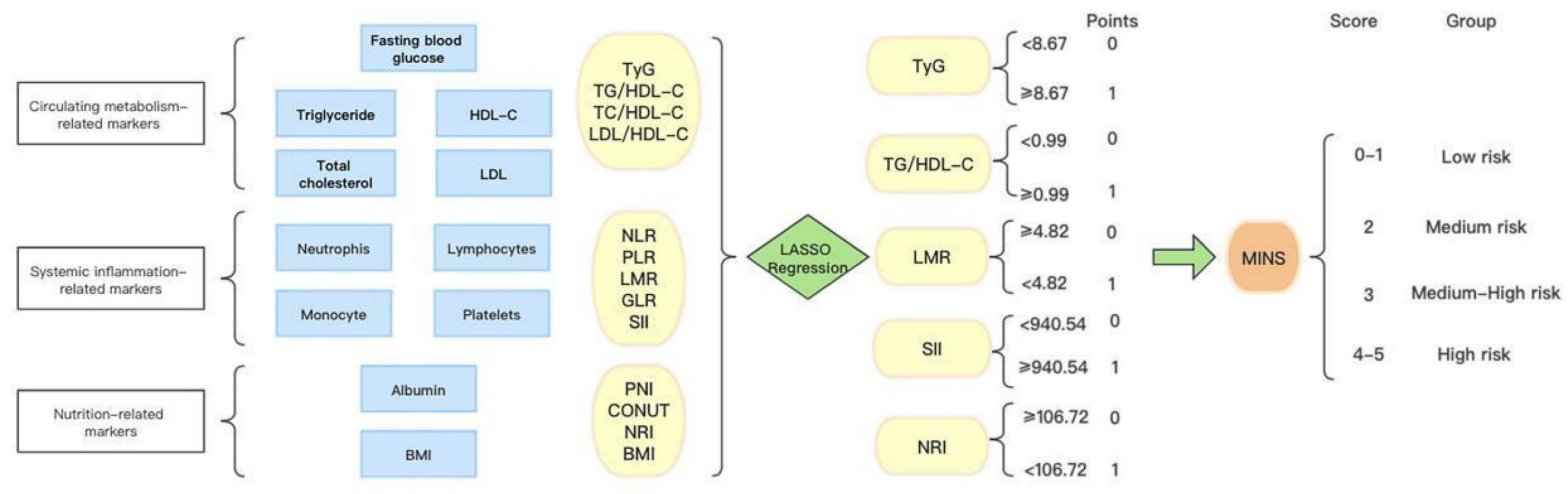
The process diagram of MINS construction and risk stratification.

**Figure 2:**
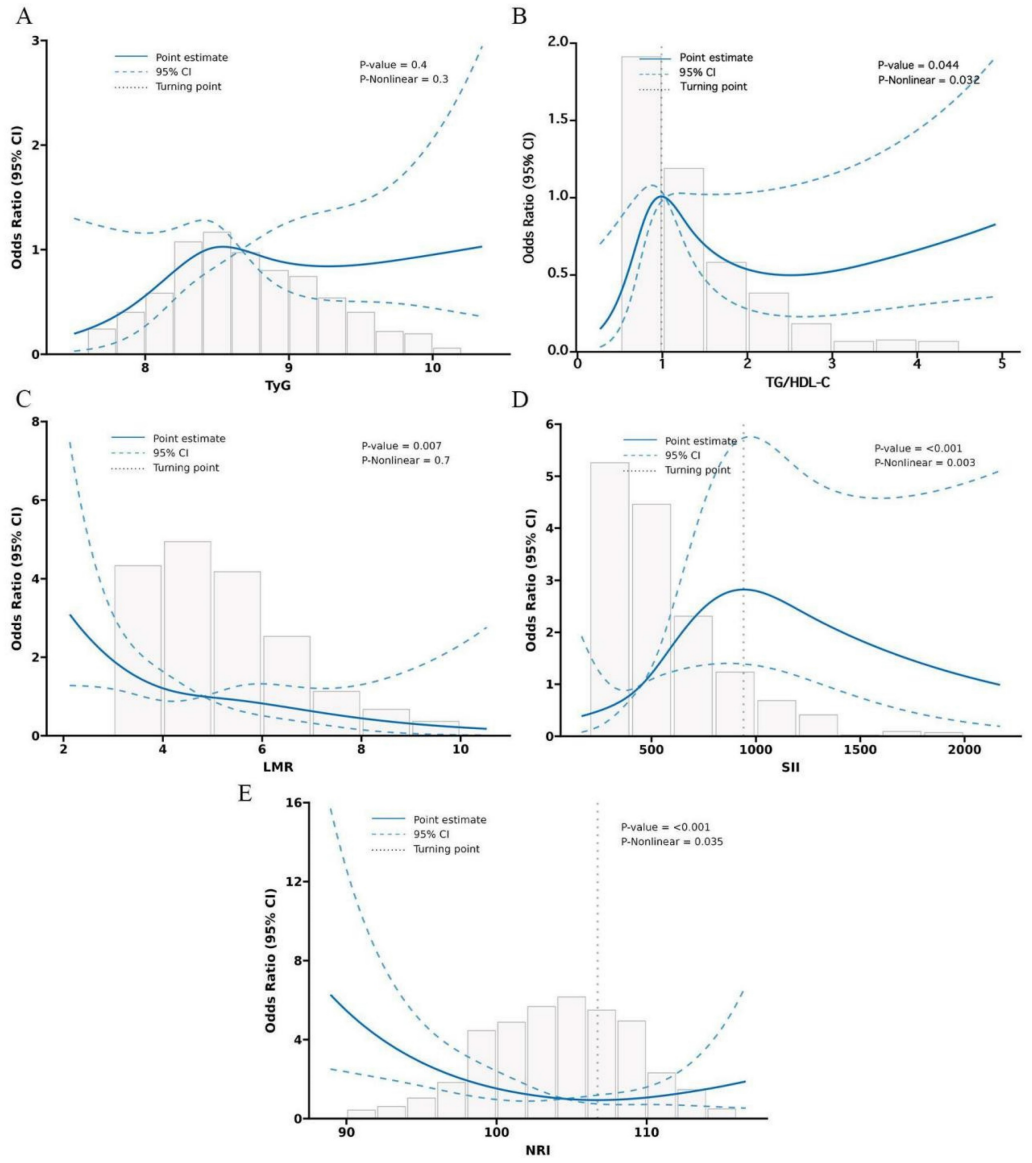
Restricted cubic spline (RCS) of 5 metabolic-inflammatory-nutritional biomarkers for predicting LNM.

In **Figure 1**, we provide a diagram showing how MINS are constructed and risk stratifications are performed. **Figure S1A** displays the correlation matrix of 13 biomarkers, with the correlation coefficients (R) ranging from -1 to 1. The multicollinearity was detected in supplement **Table S2**. According to the LASSO regression model, five features with non-zero coefficients including TyG, TG/HDL-C, SII, LMR, and NRI were selected out of the initial 13 parameters, which corresponded to the optimal value lambda.min = 0.007 (**Figures S1B and S1C**). We used RCS analyses to explore the shape of the association between LNM and each biomarker. RCS analyses showed nonlinear relationships of TG/HDL-C, SII and NRI with LNM among EC patients (all p-Nonlinear < 0.05). Subsequently, RCS curves were generated to identify the optimal cutoffs for the TG/HDL-C (0.99), SII (940.54) and NRI (106.72), as shown in** Figure 2B, 2D, and 2E**. No such nonlinear relationships were found for TyG and LMR (all p-Nonlinear > 0.05) with LNM, as depicted in **Figure 2A and 2C**. The median values for TyG and LMR were determined to be 8.67 (range 8.32-9.11) and 4.82 (range 3.76-6.00), respectively. Cutoffs of 8.67 and 4.82 were identified for the TyG and LMR, respectively. To construct the MINS, we assigned a score of 1 to high values of TyG (≥8.67), high TG/HDL-C (≥0.99), high SII (≥940.54), low LMR (<4.82), and low PRI (<106.72). All other values were scored as 0. The final MINS ranged from 0 to 5, based on the individual scores obtained.

### 3.3. Risk factors for LNM

Univariate and multivariate logistic regression analyses were conducted to analyze the association between LNM and various clinical characteristics, as summarized in **Table 1**. In the univariate analysis, potential risk factors for LNM were evaluated. After adjusting for age at diagnosis, BMI and family history of LSAC, the multivariate analysis revealed that several independent risk factors were significantly associated with LNM. These included MINS group, histological type, grade, CSI, MI and LVSI (all adjusted p<0.05).

### 3.4. Relationship of MINS group with the Clinical characteristics

The correlation between the MINS group and clinical characteristics was analyzed and presented in **Table 2**. The MINS group significantly correlated with BMI (p=0.004), CA125 (p=0.036), FIGO stage (p=0.016) and LNM (p=0.013). Additionally, the distribution of family history of LSAC, histological type and tumor grade across different MINS groups was also presented, but no significant relationship was observed between MINS and these clinical characteristics. Notably, our findings demonstrated that patients with overweight/obesity (≥23), elevated levels of the tumor marker CA125, and presence of LNM had a higher incidence in the MINS Medium-High/High Risk group (MINS 3-5).

### 3.5. Survival Analysis Based on Individual Biomarkers and MINS Groups

**Figure 3:**
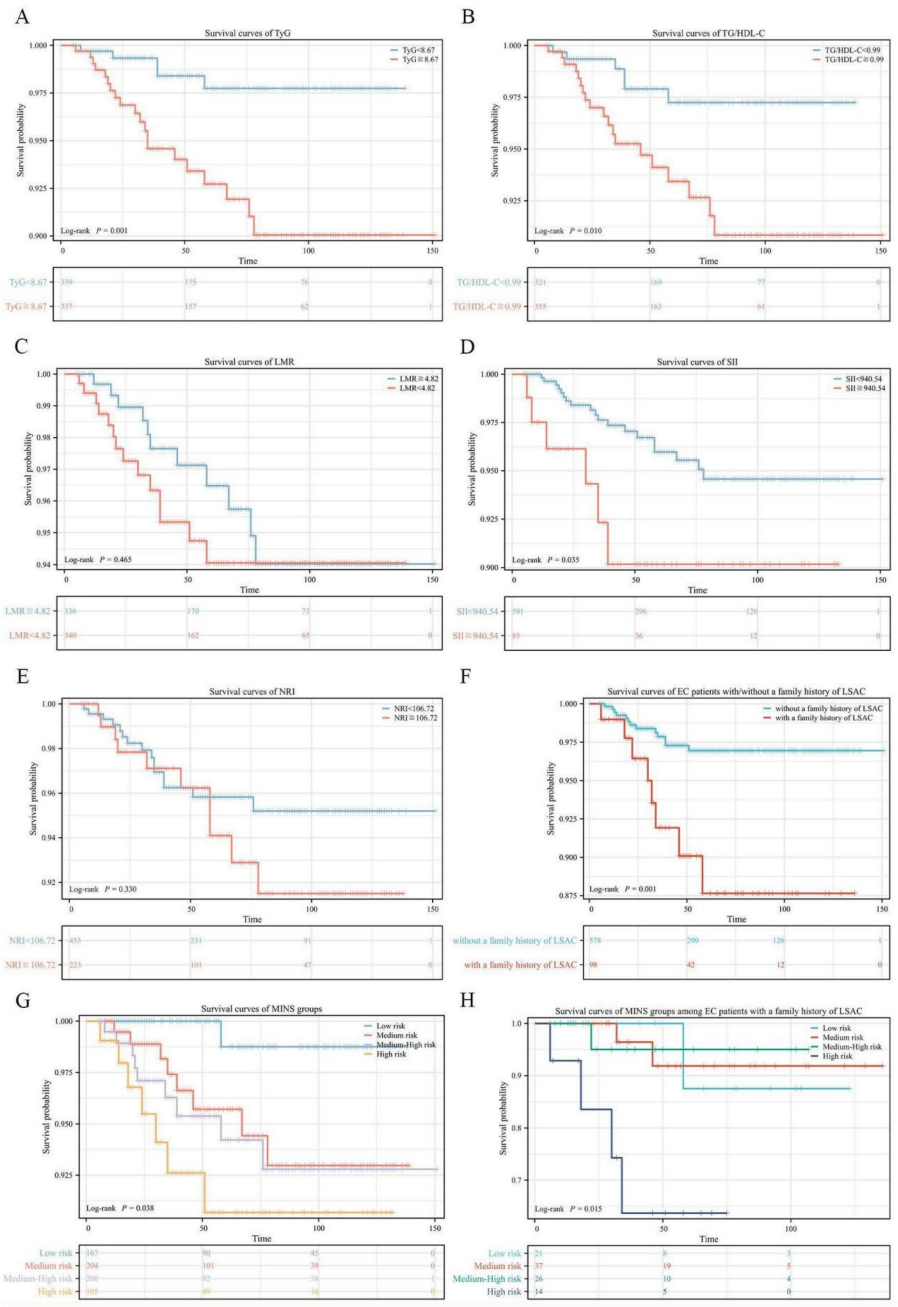
Survival curves via Kaplan-Meier analysis. (A) Survival curves of TyG. (B)Survival curves of TG/HDL-C. (C) Survival curves of LMR. (D) Survival curves of SII. (E) Survival curves of NRI. (F) Survival curves of EC patients with/without a family history of LSAC. (G) Survival curves of MINS groups. (H) Survival curves of MINS groups among EC patients with a family history of LSAC.

We compared the survival curves of each risk stratification of the MINS system (**Figure 3A-E**). Our results indicated that high TyG, high TG/HDL-C and high SII were significantly associated with shorter OS (p value=0.001; p value=0.010; p value=0.035, respectively), but no statistically significant associations of LMR or NRI with OS were observed (all p value>0.05). In **Figure 3F**, EC patients with a family history of LSAC have worse OS (p=0.001).The survival rate of patients with different MINS groups showed a stepwise decline (p=0.038), as illustrated in **Figure 3G**. OS rates of the patients in the Low, Medium, Medium-High, and High Risk groups were 99.4%, 96.1%, 95.5%, and 93.3%, respectively (Low-Medium Risk vs Low Risk: HR=7.11, 95% CI: 1.92-26.32, p=0.030; Medium-High risk vs Low risk: HR = 8.14, 95% CI: 2.35-28.16, p=0.017; High risk vs Low risk: HR = 12.32, 95% CI: 2.91-52.09, p=0.003). In EC patients with a family history of LSAC, there was a corresponding trend toward decreased survival in the different MINS groups (p=0.015), as illustrated in **Figure 3H**.

### 3.6. Construction of a Novel Prognostic Nomogram Based on MINS

**Figure 4:**
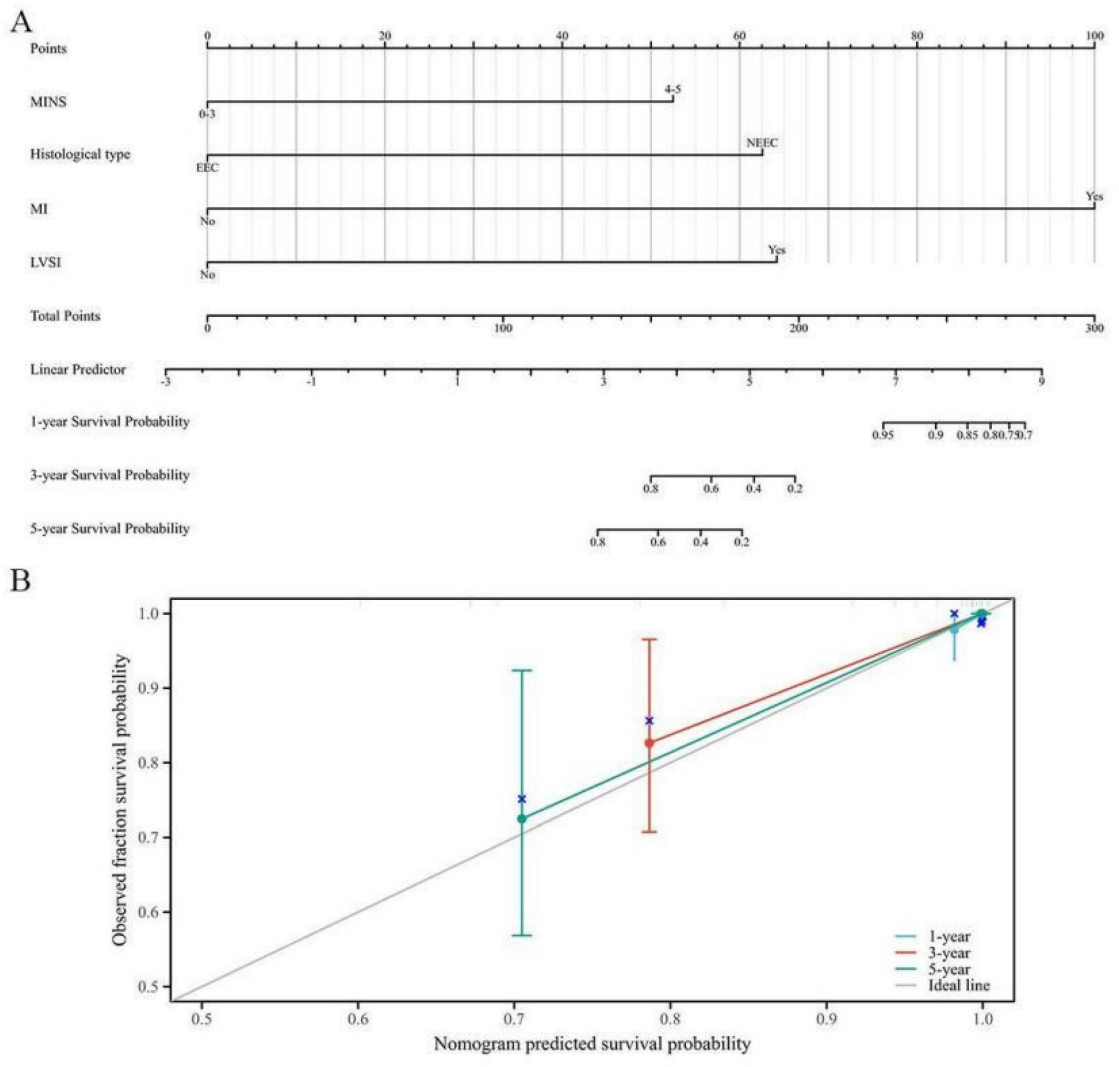
A novel prognostic nomogram based on MINS for EC patients with a family history of LSAC. (A) The nomogram for predicting 1-, 3- and 5-year survival probability in EC patients. (B) Calibration plots of the nomogram for 1-year, 3-year and 5-year.

In **Table S4**, the results of Cox Proportional-Hazards regression analysis for OS among EC patients with a family history of LSAC were shown. The multivariate Cox regression analysis revealed that MINS, histological type, MI and LVSI were the influential predictors of OS. These influential predictors were then used to create a nomogram for OS, as shown in **Figure 4A**. The prognostic nomogram demonstrated better discriminative ability, with a commendable C-index of 0.778 (95% CI: 0.725-0.832). Additionally, the calibration curves displayed satisfactory consistency in probability between the predictions from the nomogram and the observed survival outcomes, as depicted in **Figure 4B**.

## 4. Discussion

Recently, serum biomarkers for predicting LNM and prognostic factors have recently received a great deal of research attention. Several previous studies have highlighted the limitations of relying solely on a single ratio for prognostic value in EC. Rong Cong et al found that combining the ratios of NLR, PLR, and MLR yields a more robust prognostic value compared to relying on any individual ratio alone among EC patients 15. In other types of cancer, some researchers and scholars have also questioned the prognostic value of a single ratio and have suggested that the combined ratio is a better prognostic indicator 20, 21. To effectively classify risks and develop personalized treatment strategies, it is crucial to identify the most optimal predictors for LNM.

The International Guidelines recommend that LS screening should be considered for all patients with newly diagnosed EC 22, 23. Identifying individuals with LS involves a complex diagnostic process that includes taking a detailed family history and conducting a combination of genetic and immunohistochemical tests. However, the current state of LS screening is inadequate. Despite the increasing amount of evidence on LSAC, only a few studies have investigated the influence of family history of LSAC on the clinical features and prognosis of endometrial cancer patients. In our study, we also assessed the relationship between family history of LSAC and the risk of LNM and OS in endometrial cancer patients. Our findings indicate that the risk of LNM is significantly associated with a family history of LSAC, and patients with a family history of LSAC have worse OS. Family history may represent shared genetic and environmental factors, as well as their interactions.

To our knowledge, this study is the first to propose a novel system to predict LNM of EC patients by combining metabolic, inflammatory and nutritional biomarkers. Our findings indicate that MINS is a reliable predictor of LNM in EC. Furthermore, our investigation identified a significant association between elevated MINS levels and unfavorable short-term OS outcomes. A prognostic nomogram was formed by combining MINS with other clinical variables, which showed better prognostic accuracy.

At present, the mechanism underlying the association between the five biomarkers or MINS and EC remains unresolved. There has been growing evidence that inflammation is responsible for the development and progression of malignancies 24. Moreover, there has been significant attention given to the intricate interplay between nutrition imbalances and the activation of inflammation in cancer. Metabolic abnormalities and nutritional imbalance are often observed in most cancer patients. In addition, obesity is well-known to be associated with increased risk and worse prognosis of several malignant tumors, including EC. Obesity is accompanied by a series of metabolic changes that in turn promote nutritional imbalance and chronic systemic inflammatory states 25, 26. Obesity results from complex interactions between metabolic abnormalities and nutritional imbalance 27.

The predictive power of the markers that make up MINS has been confirmed in several cancers. Dyslipidemia, which mainly consists of elevated TG and lowered HDL-C, can maintain the microenvironment of inflammation through circulating metabolism and inflammatory mediators 28. A previous study by our team demonstrated that lipid reprogramming induced by the transcription factor EB (TFEB)-Estrogen-related receptor α (ERRα) axis promotes progression of EC 29. Research has shown that TG have been linked to cancer cell proliferation and differentiation as well as cancer-associated cell growth 30. In a previous study, TyG and TG/HDL-C are considered the marker of peripheral insulin resistance (IR) 31. IR is independent risk factors for the development of EC. In addition, a recent retrospective study reported that high TyG index was positively correlated with advanced pathological stages and poorer differentiation 16. Up-regulated TG and down-regulated HDL-C are strongly associated with oxidative stress and chronic inflammation and have pro-carcinogenic effects 32. Divya Seth et al. found a positive correlation between TG/HDL-C and EC risk 33. Increasing evidence have demonstrated that EC patients with more advanced tumor stages or aggressive histological types exhibit elevated TG/HDL-C levels 17. LMR is thought to reflect a balance between the favorable prognostic effects of lymphocytes and the unfavorable role of monocytes in cancer progression 14. The lower the monocyte count and the higher the lymphocyte count, the better the prognosis for both type I and type II ECs 34. Consistent with our previous report, SII serves as a valuable independent prognostic factor for LNM among EC patients 18. As a comprehensive index based on peripheral lymphocyte count, neutrophil count, and platelet count, the function of the three cells and their close relationship with the tumor could elucidate the predictive value of SII for LNM in EC patients. Elevated preoperative SII reflects a state of elevated neutrophil and platelet counts, which indicate elevated levels of inflammation in the cancer patient. Neutrophils enhance cancer cell invasion, proliferation, and metastasis 35. There is accumulating evidence to support the concept that platelets promote tumor cell proliferation and metastasis through direct interactions and the secretion of bioactive proteins 36. Low lymphocyte counts may reflect impaired host immune surveillance and also portend a poor prognosis for cancer patients 37. No study has yet been reported on the role of NRI in the clinical outcome of EC patients. As tumors grow and metastasize, they also influence and are affected by nutrient distribution in the body 38. A study conducted on elderly colorectal cancer patients found that a low preoperative NRI was associated with a poor prognosis and increased postoperative complications 39. NRI have also been reported as survival predictive marker candidates for patients with breast cancer undergoing neoadjuvant chemotherapy 40.

According to the available evidence, the current data suggests that the MINS may hold potential value as an additional prognostic tool to assist in clinical decision-making for the management of EC, both in surgical and adjuvant therapeutic settings. The major strength of this study on EC is the comprehensive consideration of metabolic characteristics, the immune-inflammatory microenvironment, and the nutritional status of the host. Integrating different factors into the model and estimating personalized risk of LNM based on each patient's characteristics usually works better than subjective judgment by clinicians. Moreover, our study aimed to inclusively incorporate all available parameters and effectively select valuable variables using the LASSO regression model, thereby reducing the potential impact of multicollinearity to some extent.

There are several potential limitations to consider. Firstly, due to the limited sample size of this study, our findings might not be generalizable to other populations. Consequently, conducting future research with a larger sample size would greatly contribute to the existing knowledge on this topic. Secondly, given the specialized obstetrics and gynecology hospital, the retrospective nature of this study made it difficult to control for potential confounders.

In conclusion, our research has shed new light on the significance of cancer-related metabolic irregularities, inflammation levels, and nutritional imbalances. Notably, we have discovered that the MINS is associated with the likelihood of LNM in patients with EC and serves as an invaluable tool for prognostic stratification among EC patients, especially those with a family history of LSAC.

## Supplementary Material

Supplementary figure and tables.

## Figures and Tables

**Table 1:** Univariate and multivariate Logistic regression analysis for predicting LNM.

		OR (univariable)	OR (multivariable)
Age at diagnosis	Mean (SD)	1.00 (0.96-1.03, p=0.867)	0.99 (0.96-1.03, p=0.763)
Menopause status	Premenopausal	-	-
	Postmenopausal	1.04 (0.60-1.81, p=0.900)	-
BMI	Mean (SD)	0.99 (0.91-1.06, p=0.728)	0.98 (0.91-1.06, p=0.615)
History of diabetes	No	-	-
	Yes	1.19 (0.59-2.25, p=0.607)	-
MINS group	Low risk	-	-
	Medium risk	3.33 (1.30-10.22, p=0.019)	3.29 (1.19-9.06, p=0.022)
	Medium-High risk	4.63 (1.87-13.97, p=0.002)	5.12 (1.88-13.89, p=0.001)
	High risk	3.04 (1.02-10.13, p=0.052)	3.26 (1.05-10.13, p=0.041)
Family history of LSAC	No		
	Yes	2.49 (1.31-4.56, p=0.004)	2.54 (1.36-4.74, p=0.003)
Histological type	EEC	-	-
	NEEC	3.29 (1.73-6.03, p<0.001)	3.71 (1.99-6.93, p<0.001)
Grade	G1-G2	-	-
	G3	3.30 (1.54-6.73, p=0.001)	3.47 (1.66-7.28, p<0.001)
CSI	No	-	-
	Yes	3.79 (2.15-6.62, p<0.001)	3.62 (2.04-6.42, p<0.001)
MI	No	-	-
	Yes	5.72 (3.27-10.31, p<0.001)	6.73 (3.65-12.42, p<0.001)
LVSI	No	-	-
	Yes	7.81 (4.39-13.91, p<0.001)	7.82 (4.36-14.04, p<0.001)

Abbreviations: BMI, Body mass index; EEC, Endometrioid endometrial cancer; NEEC, Non-endometrioid endometrial cancer; CSI, Cervical stromal invasion; MI, Myometrial invasion; LVSI, Lymph-vascular space invasion; LSAC, Lynch syndrome-associated cancer. Note: Estimates were assessed by multivariable logistics model adjusted for age at diagnosis, BMI and Family history of LSAC.

**Table 2:** Association between the MINS groups and clinical characteristics.

Characteristics	Low risk (MINS 0-1)	Medium risk (MINS 2)	Medium-High risk (MINS 3)	High risk (MINS 4-5)	p value
n	167	204	200	105	
Age at diagnosis, median (IQR)	54 (50, 58.5)	54 (49, 59.25)	55 (50, 60)	53 (49, 59)	0.520
Menopause status, n (%)					0.064
Premenopausal	62 (37.1%)	92 (45.1%)	77 (38.5%)	54 (51.4%)	
Postmenopausal	105 (62.9%)	112 (54.9%)	123 (61.5%)	51 (48.6%)	
BMI, n (%)					0.004
≥ 23	93 (55.7%)	128 (62.7%)	141 (70.5%)	78 (74.3%)	
<23	74 (44.3%)	76 (37.3%)	59 (29.5%)	27 (25.7%)	
History of diabetes, n (%)					0.126
Yes	21 (12.6%)	37 (18.1%)	44 (22%)	21 (20%)	
No	146 (87.4%)	167 (81.9%)	156 (78%)	84 (80%)	
Family history of LSAC					0.368
No	146 (87.4%)	167 (81.9%)	174 (87%)	91 (86.7%)	
Yes	21 (12.6%)	37 (18.1%)	26 (13%)	14 (13.3%)	
Histological type, n (%)					0.386
EEC	141 (84.4%)	178 (87.3%)	180 (90%)	89 (84.8%)	
NEEC	26 (15.6%)	26 (12.7%)	20 (10%)	16 (15.2%)	
FIGO stage, n (%)					0.016
I-II	155 (92.8%)	171 (83.8%)	163 (81.5%)	90 (85.7%)	
III-IV	12 (7.2%)	33 (16.2%)	37 (18.5%)	15 (14.3%)	
Grade, n (%)					0.807
G1-G2	119 (86.9%)	142 (87.1%)	155 (88.1%)	72 (83.7%)	
G3	18 (13.1%)	21 (12.9%)	21 (11.9%)	14 (16.3%)	
MI, n (%)					0.399
No	125 (74.9%)	144 (70.6%)	146 (73%)	69 (65.7%)	
Yes	42 (25.1%)	60 (29.4%)	54 (27%)	36 (34.3%)	
LVSI, n (%)					0.475
No	146 (87.4%)	174 (85.3%)	176 (88%)	86 (81.9%)	
Yes	21 (12.6%)	30 (14.7%)	24 (12%)	19 (18.1%)	
LNM, n (%)					0.013
No	162 (97%)	185 (90.7%)	175 (87.5%)	96 (91.4%)	
Yes	5 (3%)	19 (9.3%)	25 (12.5%)	9 (8.6%)	

Abbreviations: EEC, Endometrioid endometrial cancer; NEEC, Non-endometrioid endometrial cancer; FIGO, International Federation of Gynecology and Obstetrics; CSI, Cervical stromal invasion; MI, Myometrial invasion; LVSI, Lymph-vascular space invasion; BMI, Body mass index; LSAC, Lynch syndrome-associated cancer.
