# Supplementary material for: A metabolic-inflammatory-nutritional score (MINS) is associated with lymph node metastasis and prognostic stratification for endometrial cancer patients
Source: Int J Med Sci. 2024 Sep 9;21(12):2379–89. doi: 10.7150/ijms.96179 (PMC11413899; doi:10.7150/ijms.96179)
Supplement: Supplementary file 1 — Supplementary figure and tables. [file ijmsv21p2379s1.pdf]

Supplementary Materials

Figure.S1.

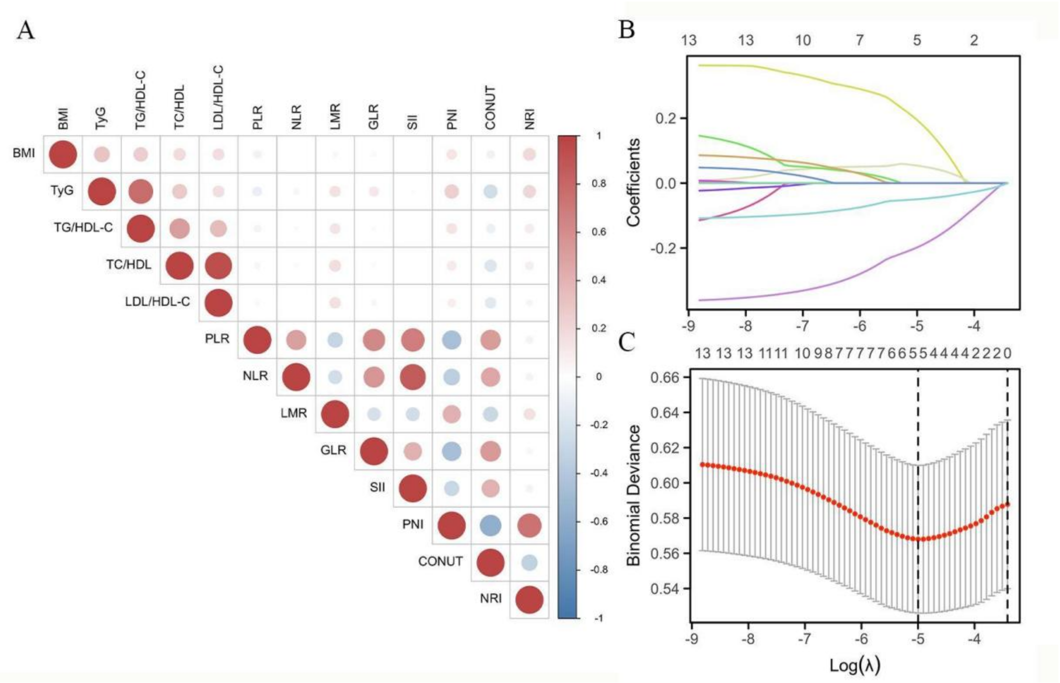

Construction of the MINS using LASSO Cox regression model. (A) A correlation matrix is represented from R=-1 negative correlation (red) to R=1 positive correlation (blue). (B) LASSO coefficient profiles of the 13 metabolic-inflammatory-nutritional biomarkers. (C) Ten-fold cross-validation for tuning parameter selection in the LASSO model.

Table S1. The baseline clinicopathological characteristics of EC patients.

Characteristics	No. of patients (%)	Metabolisc, Inflammatory, and Nutritional indicators	No. of patient s (%)
Age at diagnosis		BMI (kg/m^2)	
Median (IQR)	54 (50, 59)	≥ 23	440 (65.1%)
Menopause status		<23	236 (34.9%)
Premenopausal	285 (42.2%)	Albumin(g/L)	
Postmenopausal	391 (57.8%)	≥ 40	455 (67.3%)
History of diabetes		< 40	221 (32.7%)
Yes	123 (18.2%)	Neutrophil count(×10^9/L)	
No	553 (81.8%)	≥ 6.3	46 (6.8%)
Family history of LSAC		< 6.3	630 (93.2%)
No	578 (85.5%)	Lymphocyte count(×10^9/L)	
Yes	98 (14.5%)	≥1.1	631 (93.3%)
Histological type		< 1.1	45 (6.7%)
EEC	588 (87.0%)	Monocyte count(×10^9/L)	
NEEC	88 (13.0%)	≥0.6	51 (7.5%)
FIGO stage		<0.6	625 (92.5%)
I-II	579 (85.7%)	Platelet count(×10^9/L)	
III-IV	97 (14.3%)	≥350	65 (9.6%)
Grade		<350	611 (90.4%)
G1-G2	488 (86.8%)	Fasting blood glucose(mmol/L)	
G3	74 (13.2%)	>6.1	144(21.3%)
Unknown	114	≤6.1	532 (78.7%)
CSI		Triglyceride(mmol/L)	
No	548 (81.1%)	≥1.7	219 (32.4%)
Yes	128 (18.9%)	<1.7	457 (67.7%)
MI		Total cholesterol(mmol/L)	
<50%	484 (71.6%)	≥5.2	304 (45.0%)
≥50%	192 (28.4%)	<5.2	372 (55.0%)
LVSI		HDL-C(mmol/L)	
No	582 (86.1%)	≥1.29	379 (56.1%)
Yes	94 (13.9%)	<1.29	297 (43.9%)
LNM		LDL (mmol/L)	
No	618 (91.4%)	≥3.10	342 (50.6%)
Yes	58 (8.6%)	<3.10	334 (49.4%)

Abbreviations: EEC Endometrioid endometrial cancer, NEEC Non-endometrioid endometrial cancer,

LSAC: Lynch syndrome-associated cancer, FIGO International Federation of Gynecology and Obstetrics,

CSI Cervical stromal invasion, MI Myometrial invasion, LVSI Lymph-vascular space invasion, BMI Body mass index, HDL-C High-density lipoprotein cholesterol, LDL Low-density lipoprotein.

Table S2. The formula of metabolic-inflammatory-nutritional biomarkers.

Indicators	The formula
MINS	high TyG (≥8.67), high TG/HDL-C (≥0.99), low LMR (<4.82), high SII (≥940.54), and low NRI (<106.72) were scored as 1, and the remaining values were scored as 0. All scores were added together, and the final risk stratification was categorized into four groups (low risk, medium risk, medium-high risk, high risk) according to the scores
BMI	weight (kg)/height^2 (m)
TyG	ln[triglyceride (TG, mg/dl)×fasting blood glucose (FBG, mg/dl)]/2
TG/HDL-C	Triglyceride (mmol/L)/High density lipoprotein cholesterol (mmol/L)
TC/HDL-C	Total cholesterol (mmol/L)/High density lipoprotein cholesterol (mmol/L)
LDL/HDL-C	Low density lipoprotein cholesterol (mmol/L)/high density lipoprotein cholesterol (mmol/L)
NLR	Neutrophil (10^9/L)/Lymphocyte (10^9/L) ratio
PLR	Platelet (10^9/L) /Lymphocyte (10^9/L) ratio
LMR	Lymphocyte (10^9/L) /monocyte (10^9/L) ratio
GLR	Glucose (mmol/l)/Lymphocyte (10^9/L) ratio
SII	Neutrophil (10^9/L) × Platelet (10^9/L)/Lymphocyte (10^9/L) ratio
PNI	Albumin (g/L) + 5×lymphocyte (10^9/L)
CONUT	(1) Albumin (g/L), ≥35 0 score;<br>Albumin (g/L), 30-34.9 2 score;<br>Albumin (g/L), 25-29.9 4 score;<br>Albumin (g/L), <25 6 score;<br>(2) Lymphocyte (×10^9/L), >1.6 0 score;<br>Lymphocyte (×10^9/L), 1.2-1.59 1 score;<br>Lymphocyte (×10^9/L), 0.8-1.19 2 score;<br>Lymphocyte (×10^9/L), <0.8 3 score;<br>(3) Total cholesterol (mmol/L), ≥4.662 0 score<br>Total cholesterol (mmol/L),3.626-4.662 1 score;<br>Total cholesterol (mmol/L),2.59-3.626 2 score;<br>Total cholesterol (mmol/L), <2.59 3 score;

NRI	1.519× albumin (g/L) + 41.7 × (body weight/ideal body weight). We set BW/IBW =1 when BW exceeded IBW. Ideal body weight was calculated using the Lorentz formula, as follows: Female, IBW = height -100-(height -150)/2.5.

Table S3. The collinearity between the independent variables.

Term	VIF	VIF_CI_low	VIF_CI_high	SE_factor	Tolerance	Tolerance_CI_low	Tolerance_CI_high
BMI	1.34	1.23	1.48	1.16	0.75	0.67	0.81
TyG	4.40	3.87	5.02	2.10	0.23	0.20	0.26
TG/HDL-C	4.98	4.38	5.70	2.23	0.20	0.18	0.23
TC/HDL-C	11.33	9.86	13.06	3.37	0.09	0.08	0.10
LDL/HDL-C	8.43	7.35	9.70	2.90	0.12	0.10	0.14
PLR	6.85	5.98	7.86	2.62	0.15	0.13	0.17
NLR	7.75	6.76	8.90	2.78	0.13	0.11	0.15
LMR	1.62	1.47	1.80	1.27	0.62	0.55	0.68
GLR	3.58	3.16	4.07	1.89	0.28	0.25	0.32
SII	12.10	10.52	13.95	3.48	0.08	0.07	0.10
PNI	7.08	6.19	8.13	2.66	0.14	0.12	0.16
CONUT	2.55	2.28	2.88	1.60	0.39	0.35	0.44
NRI	5.00	4.39	5.72	2.24	0.20	0.17	0.23

Table S4. Results of Univariate and multivariate Cox regression analysis of OS among EC patients with a family history of LSAC

Characteristic	Univariable			Multivariable		
	HR	95% CI	**p-value**	HR	95% CI	**p-value**
MINS						
0-3	-	-		-	-	
4-5	7.07	1.77, 28.30	0.006	8.84	1.02, 50.12	0.020
Age at diagnosis	1.05	0.95, 1.15	0.357			
BMI	0.85	0.68, 1.05	0.134			
Histological type						
EEC	-	-		-	-	
NEEC	15.77	3.78, 65.74	<0.001	0.079	0.01，0.69	0.022
Grade						
G1-2	-	-				
G3	5.64	0.79, 40.04	0.084			
CSI						
No	-	-				
Yes	0.83	0.17, 4.14	0.825			
MI						
No	-	-		-	-	
Yes	15.9	1.95, 129.41	0.01	57.40	2.53, 1302.87	0.011
LVSI						
No	-	-		-	-	
Yes	12.32	2.91, 52.29	<0.001	13.457	2.00, 90.49	0.008

Abbreviations: BMI, Body mass index; EEC, Endometrioid endometrial cancer; NEEC, Non-endometrioid endometrial cancer; CSI, Cervical stromal invasion; MI, Myometrial invasion; LVSI, Lymph-vascular space invasion
